# Supplementary material for: Evolving public behavior and attitudes towards COVID-19 and face masks in Taiwan: A social media study
Source: PLoS One. 2021 May 20;16(5):e0251845. doi: 10.1371/journal.pone.0251845 (PMC8136722; doi:10.1371/journal.pone.0251845)
Supplement: S1 Appendix — (DOCX) [file pone.0251845.s001.docx]

S1 Appendix. Information about the scope of the data and KEYPO big data analytics engine.

Given that the study used a web-based methodology to capture public epidemic awareness of COVID-19 through collecting social media- and Internet-based data, the study adopted KEYPO Big Data Analytics Engine, which is well-known online public opinion system in Taiwan, to collect online data. Many Taiwanese official departments and researchers use the KEYPO big data system as a research tool to analyze the Internet opinion and sentiment (e.g., Ku & Liu, 2019; Chin, Tang, & Lee, 2020). This appendix elaborates how we used KEYPO as a research tool to conduct the big data analysis. The following sections will introduce the research subjects, data source, the time range of data, keywords for analysis, and techniques for retrieving data.

1. Research subjects and data source

In the field of opinion research, using big data analysis on the data volume on the Internet to capture public opinion has become a new paradigm. Compared to a traditional opinion survey, the study focused on the pieces of data left by Internet users, rather than on individuals’ responses collected from questionnaires. Specifically, the online text data on the Internet is the focus of the study. It mainly covers online articles and the comments attached to the articles. Regarding the data source, the web crawler program, KEYPO, covers over 10,000 internet channels in Taiwan on social network sites (e.g., Facebook, Instagram, YouTube), blogs (e.g., Pixnet, Xuite, Blogger), Internet forums (e.g., Dcard, PTT, Mobile01), and news websites (e.g., SETN, CAN, ETToday). KEYPO had collected 20 billion data records by 2020, and the amount of data continues to grow at the rate of one million records per day.

In the study, we used KEYPO to collect online text data related to “COVID-19” and “face masks” from all included channels on the Internet from December 31, 2019, to February 29, 2020. During that period, the total number of mentions of COVID-19 and face masks reached 3,661,292 and 3,326,528 data records, respectively. These two data were used to construct the time series variables termed as “volume of mentions of COVID-19 on social media” and “volume of mentions of face masks on social media.” On the other hand, the data of the time series variables of “Number of news reports on COVID-19” were collected from the news channels.

1. Keywords for analysis

Mandarin (Traditional Chinese), the official language of Taiwan, was used in the study. There were two different names to represent COVID-19 in the early stage in Taiwan, namely “武漢肺炎(Wuhan coronavirus pneumonia)” and “新冠肺炎(new coronavirus pneumonia).” Therefore, the keywords “(武漢|新冠)&肺炎” were used in the KEYPO search engine. On the other hand, the Mandarin for face masks is “口罩”, which was used in the KEYPO search engine.

1. More details about the data retrieval

The KEYPO crawler program mainly retrieves the title, author, content, publication date, publication source, and community feedback (such as being liked, being shared, being commented on, etc.) and the link URL field in the crawling webpage. For those that provide an API interface, KEYPO uses this API to obtain the above information in a formal manner that conforms to the norms; for the websites without API interfaces, KEYPO simulates web browsing behaviors and uses automated web crawlers to grab the data.

Due to the different privacy policies of each platform, KEYPO follows the norms of each platform and provides authorized results. KEYPO obeys the privacy policy and does not analyze personal data. This research believes that crawling the publicly known information on the Internet and conducting a text analysis meets the purpose of being publicly known.

**References**

Chin, C., Tang, C., & Lee, Y. (2020). The social network volume of COVID-19 and stock market response. 10(6), 77–97.

Ku, Y.-C., & Liu, P.-Y. (2019). Internet opinion and sentiment of the prosecutorial system: A big-data analysis. Academy for the Judiciary, Ministry of Justice in Taiwan. Retrieved from <https://www.tpi.moj.gov.tw/291066/291069/291101/291109/646158/post>
