# Supplementary material for: Evolving public behavior and attitudes towards COVID-19 and face masks in Taiwan: A social media study
Source: PLoS One. 2021 May 20;16(5):e0251845. doi: 10.1371/journal.pone.0251845 (PMC8136722; doi:10.1371/journal.pone.0251845)
Supplement: S2 Appendix — (DOCX) [file pone.0251845.s002.docx]

**S2 Appendix. The output of the autocorrelation function (ACF) and partial autocorrelation function (PACF).**

This appendix provides the results of the autocorrelation function (ACF) and partial autocorrelation function (PACF). The main purpose of using ACF and PACF is to examine whether any over-time correlation exists in its residuals. The output of ACF indicates that all six variables, namely total confirmed cases of COVID-19 in Taiwan, number of news reports on COVID-19, the volume of mentions of COVID-19 on social media, the volume of mentions of face masks on social media, Google search volume of COVID-19, and Google search volume of face masks, have significantly autocorrelative relationships, which would cause spurious relationships in our further time series analysis.

The output of PACF indicates that all six variables have autocorrelation at the first lag only with the significant peak at lag 1. This is often called an autoregressive process at one lag [AR(1) process]. Given that, the first-order autocorrelation would be removed in the cross-correlation.

| Autocorrelation function | Partial autocorrelation function |
| --- | --- |
| Total confirmed cases of COVID-19 in Taiwan | |
| 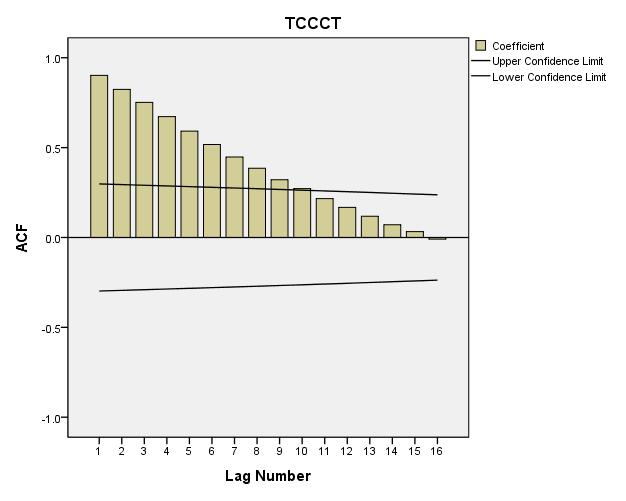 | 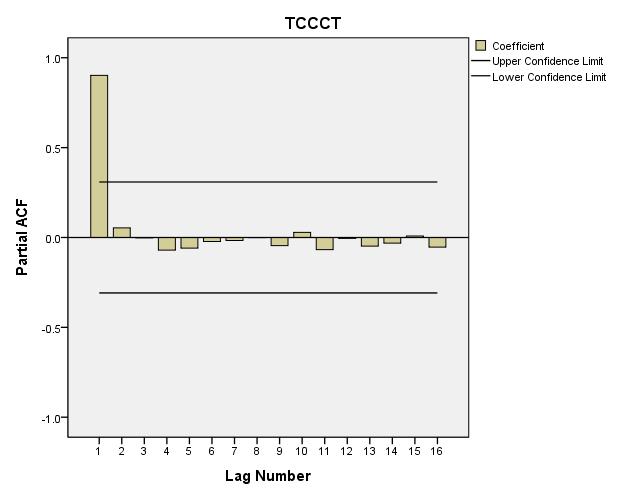 |
| Number of news reports on COVID-19 | |
| 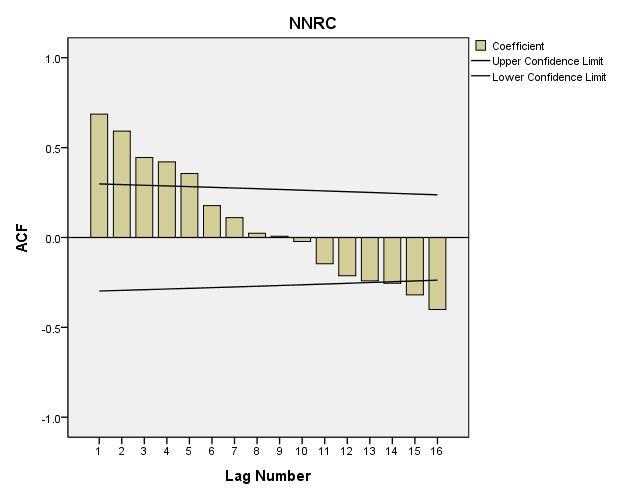 | 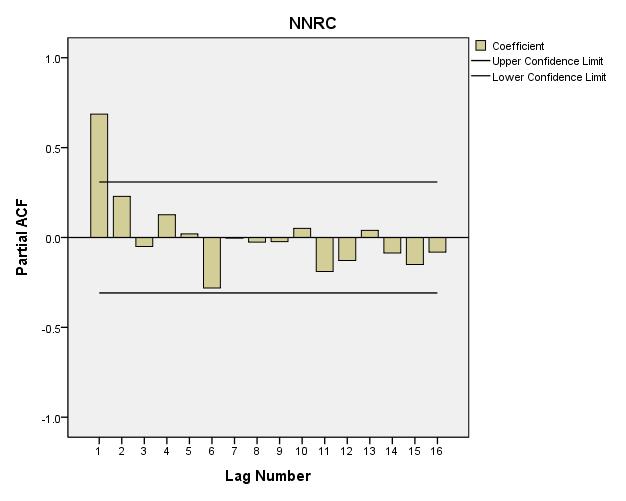 |
| Volume of mentions of COVID-19 on social media | |
| 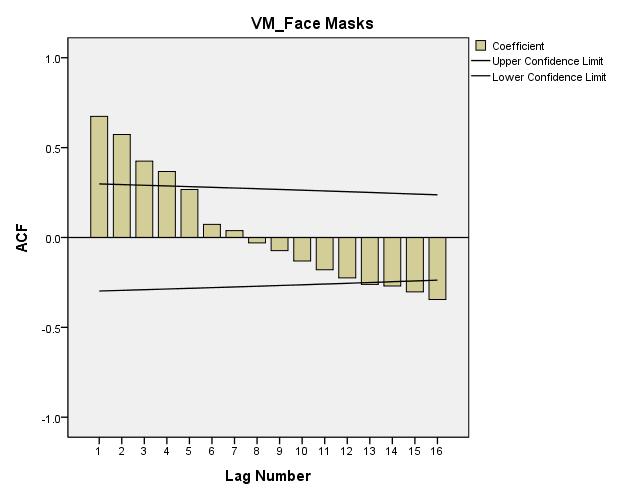 | 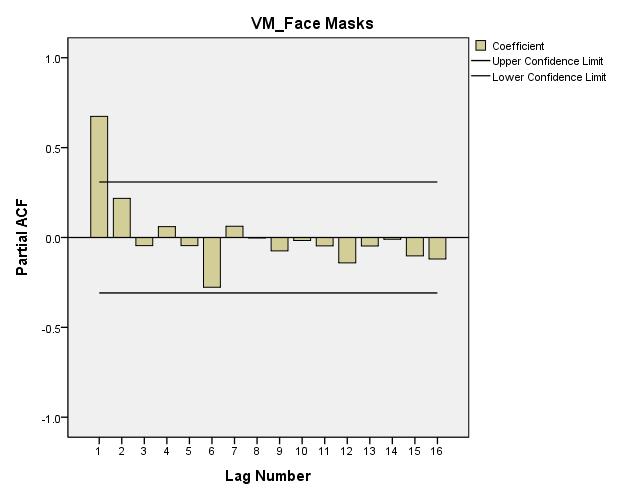 |
| Volume of mentions of face masks on social media | |
| 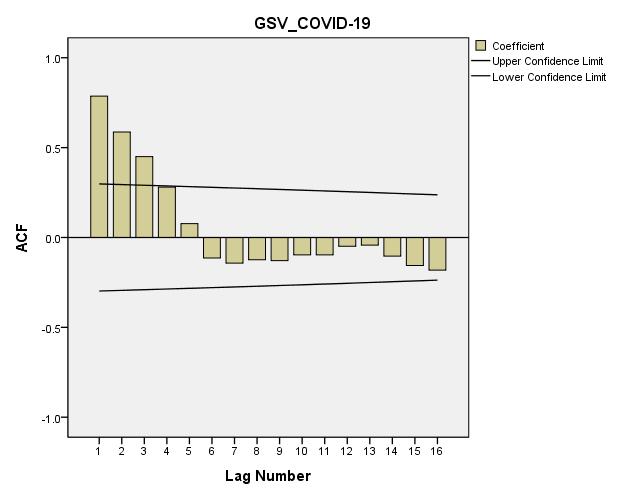 | 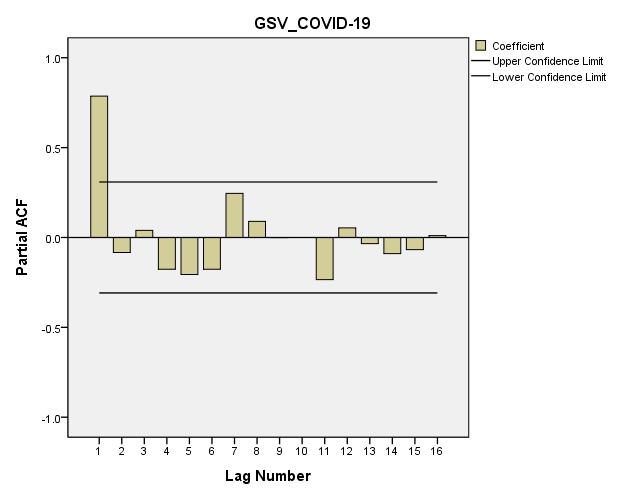 |
| Google search volume of COVID-19 | |
| 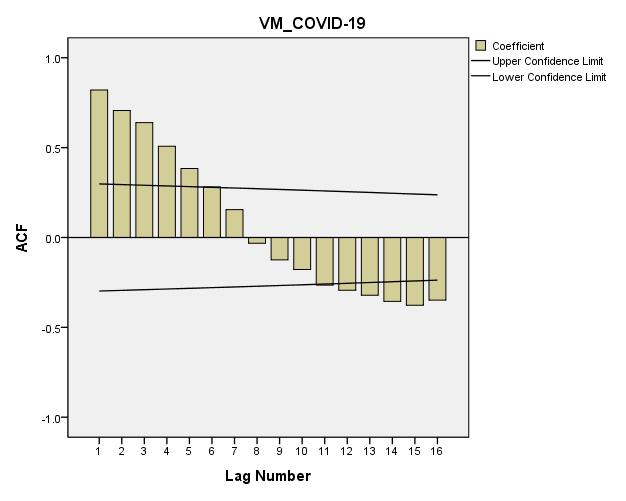 | 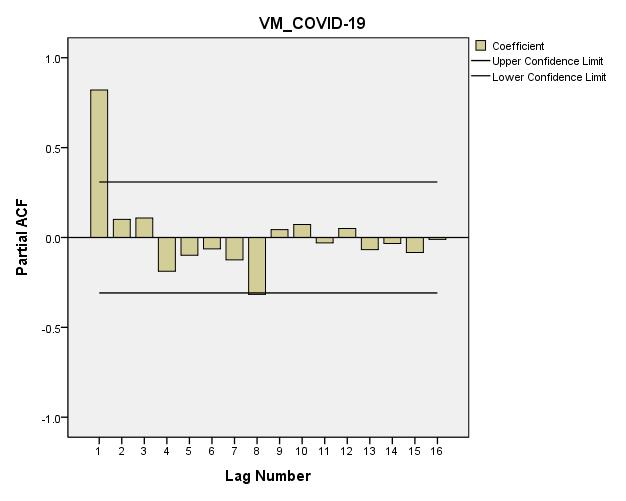 |
| Google search volume of face masks | |
| 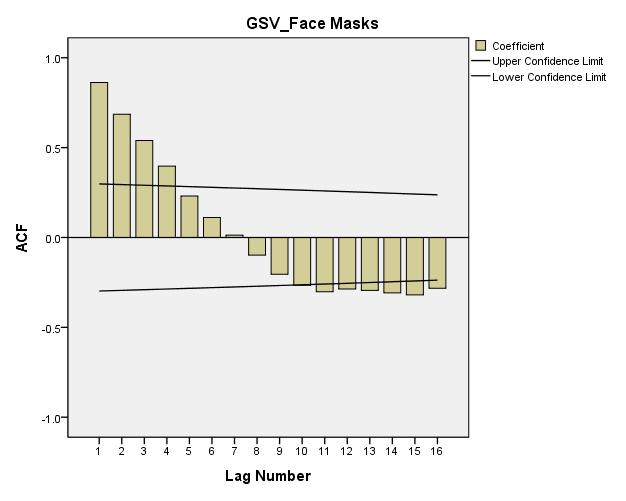 | 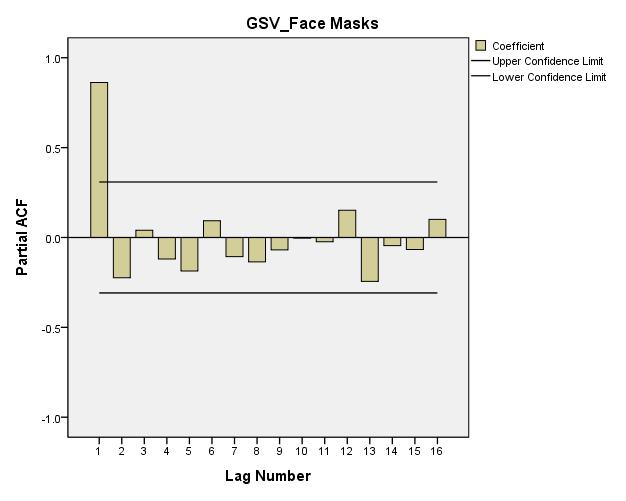 |
