# Supplementary material for: Evolving public behavior and attitudes towards COVID-19 and face masks in Taiwan: A social media study
Source: PLoS One. 2021 May 20;16(5):e0251845. doi: 10.1371/journal.pone.0251845 (PMC8136722; doi:10.1371/journal.pone.0251845)
Supplement: S3 Appendix — (DOCX) [file pone.0251845.s003.docx]

**S3 Appendix. The output of the cross-correlation function (CCF) after whitening the data by removing the first-order autocorrelation.**

This appendix provides complete output of cross correlation function with day time lags between -7 and 7 among the six variables, including total confirmed cases of COVID-19 in Taiwan, number of news reports on COVID-19, the volume of mentions of COVID-19 on social media, the volume of mentions of face masks on social media, Google search volume of COVID-19, and Google search volume of face masks.

| Total number of confirmed cases of COVID-19 in Taiwan with Number of news reports on COVID-19 | Total number of confirmed cases of COVID-19 in Taiwan with Volume of mentions of COVID-19 on social media |
| --- | --- |
| 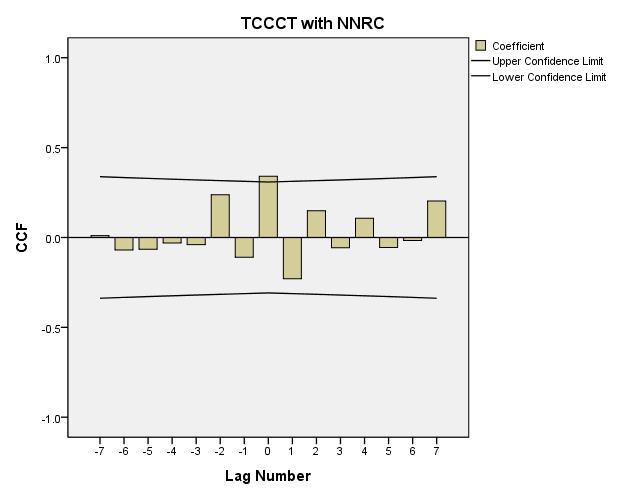 | 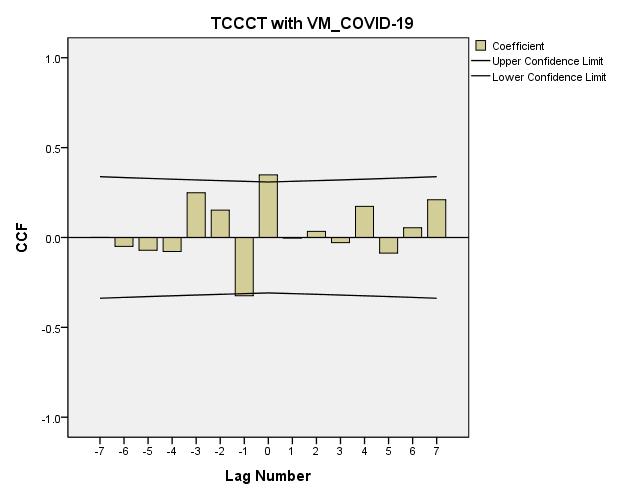 |
| Total number of confirmed cases of COVID-19 in Taiwan with Volume of mentions of face masks on social media | Total number of confirmed cases of COVID-19 in Taiwan with Google search volume for COVID-19 |
| 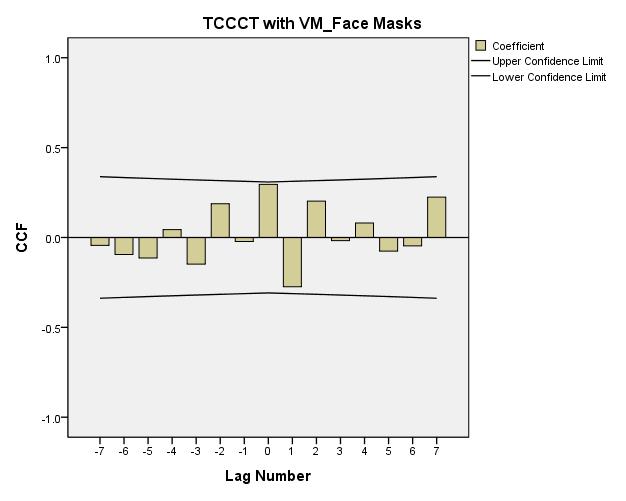 | 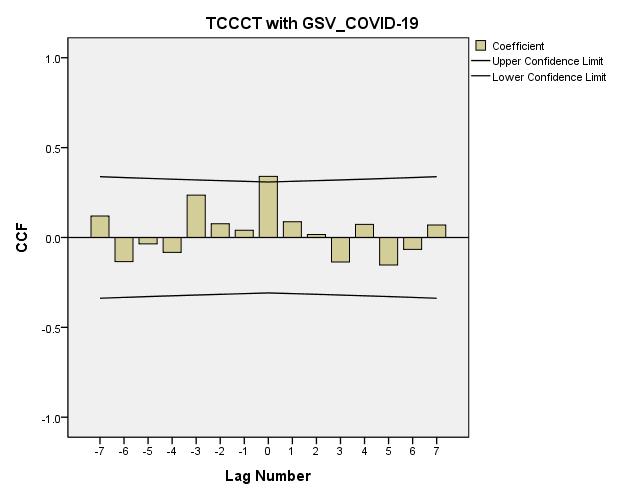 |
| Total number of confirmed cases of COVID-19 in Taiwan with Google search volume for face masks | Number of news reports on COVID-19 with Volume of mentions of COVID-19 on social media |
| 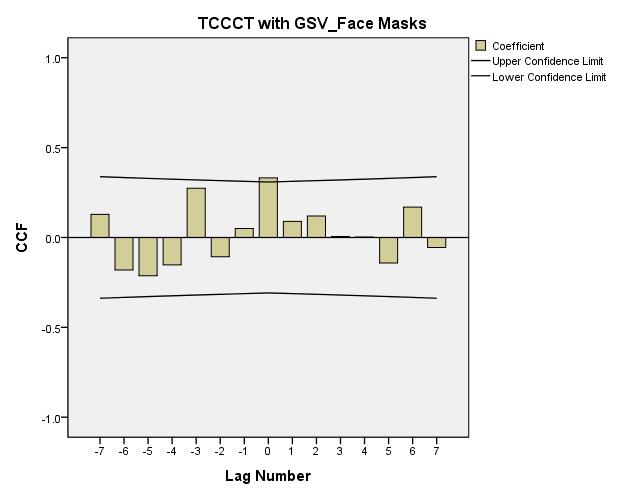 | 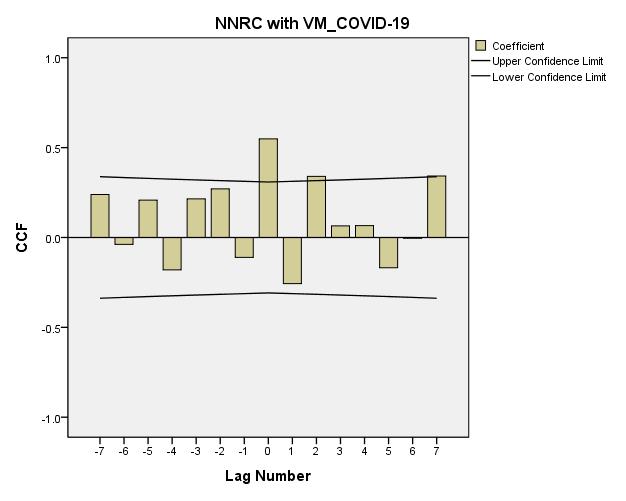 |
| Number of news reports on COVID-19 with Volume of mentions of face masks on social media | Number of news reports on COVID-19 with Google search volume for COVID-19 |
| 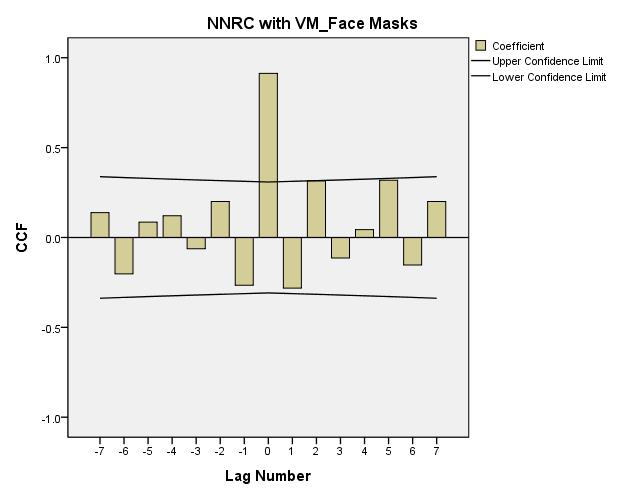 | 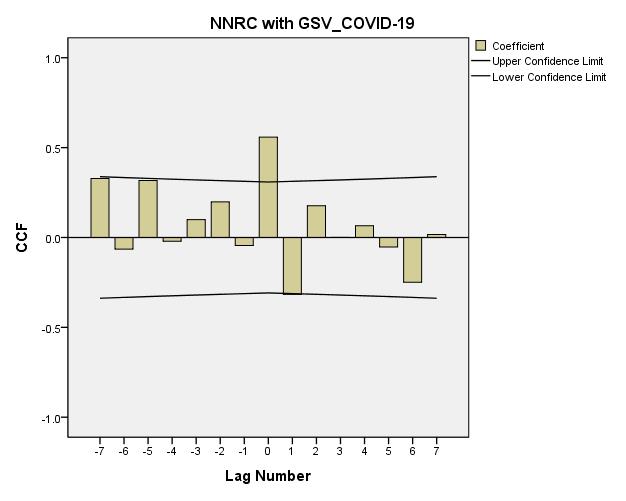 |
| Number of news reports on COVID-19 with Google search volume for face masks | Volume of mentions of COVID-19 with Volume of mentions for face masks |
| 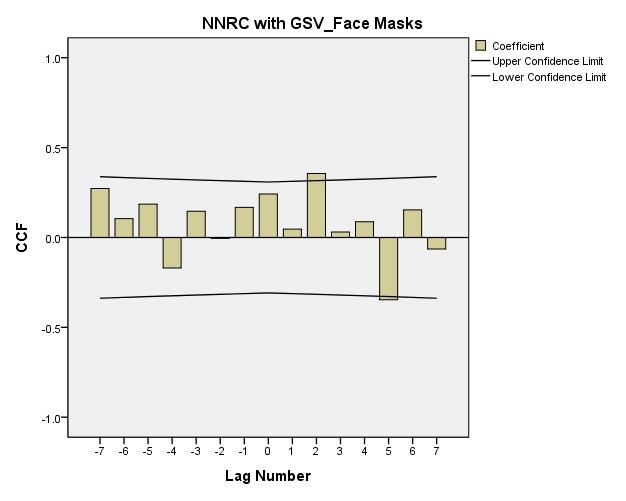 | 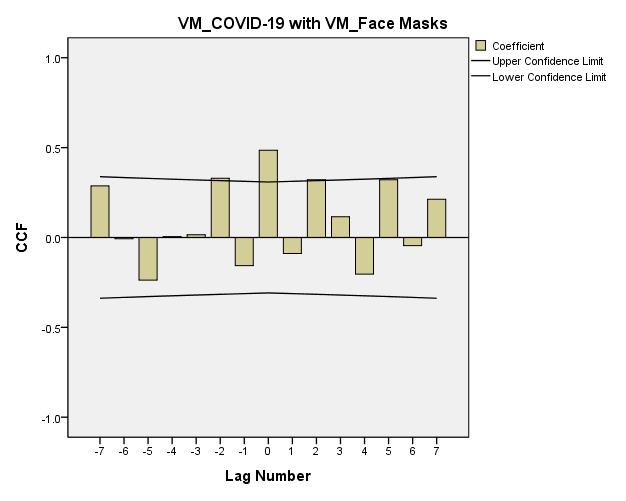 |
| Volume of mentions of COVID-19 with Google search volume for COVID-19 | Volume of mentions of COVID-19 with Google search volume for face masks |
| 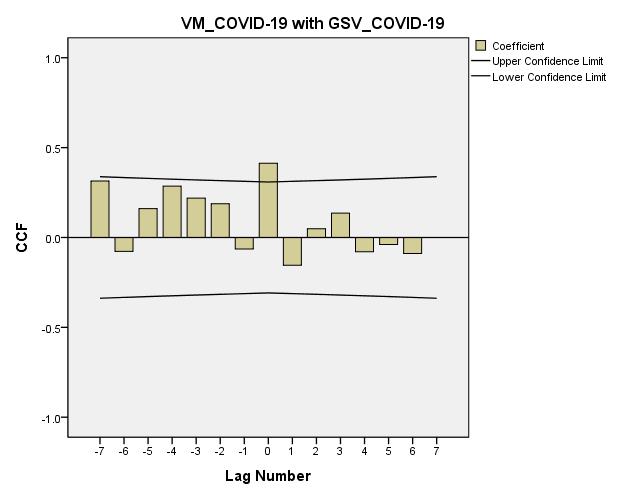 | 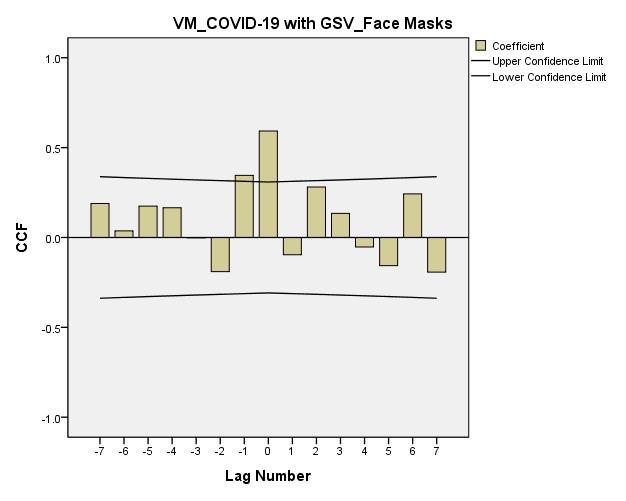 |
| Volume of mentions of face masks with Google search volume for COVID-19 | Volume of mentions of face masks with Google search volume for face masks |
| 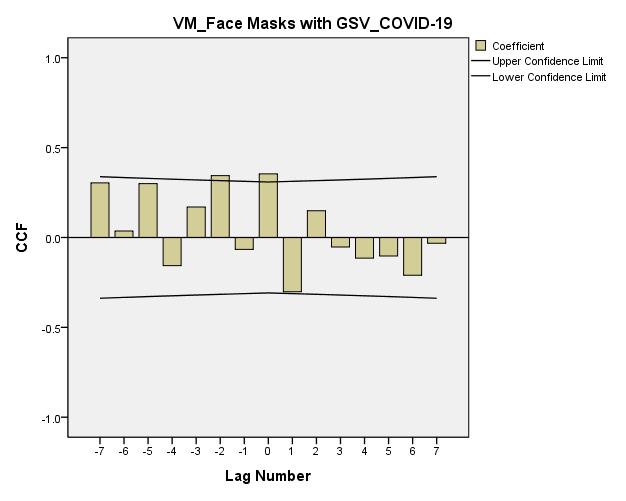 | 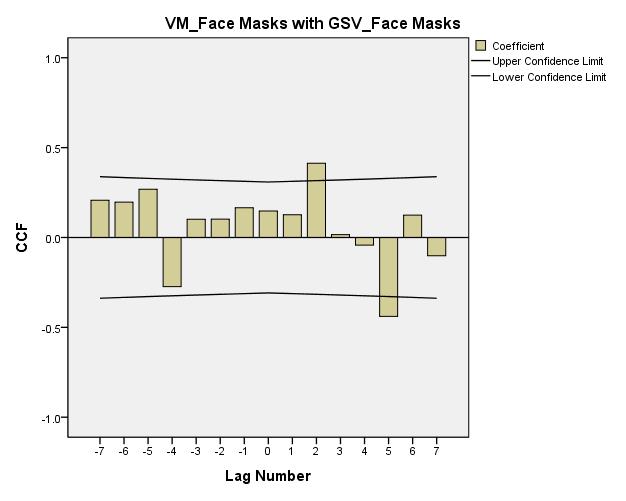 |
| Google search volume for COVID-19 with Google search volume for COVID-19 | |
| 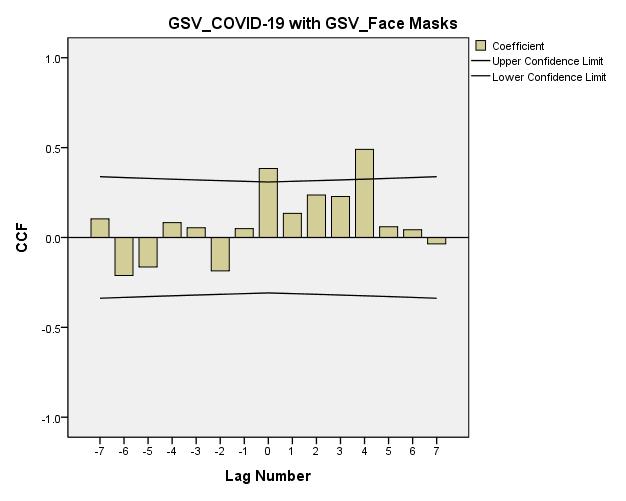 | |
