# Supplementary material for: Evolving public behavior and attitudes towards COVID-19 and face masks in Taiwan: A social media study
Source: PLoS One. 2021 May 20;16(5):e0251845. doi: 10.1371/journal.pone.0251845 (PMC8136722; doi:10.1371/journal.pone.0251845)
Supplement: S1 Table — (DOCX) [file pone.0251845.s007.docx]

**S1 Table. The categorization of the types of fear**

| **Types of fear** | **Explanation** | **Examples (Abstract of events)** |
| --- | --- | --- |
| Mistrust | Referring to the people, government, or corporate institutions exposing us to risk in the first place, or the distrust of people who are supposed to protect us. | - Case number 25:   Suspected COVID-19 epidemic concealed   - Case number 143:   Questioning the government’s negligence of home isolation and quarantine management policies |
| Severity | Referring to the seriousness of the condition on an individual’s health and safety. | - Case number 115:   First confirmed death from COVID-19 in Taiwan   - Case number 136:   Recovered patients have partially reduced lung function: The after effect of pulmonary fibrosis |
| Loss of control | Referring to one’s inability to take control of self-protection or to live. | - Case number 67:   Shortages of face masks supplies   - Case number 84:   feeling uneasy about buying masks, toilet paper, and other protective and essential supplies |
| Uncertainty | Referring to the current ignorance or ambiguity of the characteristics of the situation of an emerging disease. | - Case number 18:   The news of the possibility of human-to-human transmission of COVID-19   - Case number 52:   The time of survival and the conditions affecting the 2019-nCoV viability in the environment are currently unknown. |
| Susceptibility | Referring to the increases in the possibility of an individual getting a disease. | - Case number 37:   The first confirmed cased of COVID-19 in Taiwan   - Case number 198:   People who did not obey the rules of home quarantine |
| Without fear | Referring to the event is not embedded with fear-arousal stimuli information | - Case number 252:   Team of Academic Sinica in Taiwan develops antibodies for rapid virus screening   - Case number 198:   The implementation of the name-based rationing system new version 2.0 |
