# Supplementary material for: Evolving public behavior and attitudes towards COVID-19 and face masks in Taiwan: A social media study
Source: PLoS One. 2021 May 20;16(5):e0251845. doi: 10.1371/journal.pone.0251845 (PMC8136722; doi:10.1371/journal.pone.0251845)
Supplement: S2 Table — (DOCX) [file pone.0251845.s008.docx]

**S2 Table. The results of one-way analysis of variance for Google search volume for face masks in different periods.**

|  | Sum of squares | *df* | Mean square | *F* | *p* |
| --- | --- | --- | --- | --- | --- |
| Between groups | 22542.060 | 2 | 11271.030 | 65.907 | < .05 |
| Within groups | 14023.251 | 82 | 171.015 |  |  |
| Total | 36565.312 | 84 |  |  |  |
