# Supplementary material for: Evolving public behavior and attitudes towards COVID-19 and face masks in Taiwan: A social media study
Source: PLoS One. 2021 May 20;16(5):e0251845. doi: 10.1371/journal.pone.0251845 (PMC8136722; doi:10.1371/journal.pone.0251845)
Supplement: S3 Table — (DOCX) [file pone.0251845.s009.docx]

**S3 Table. The results of post hoc multiple comparisons for Google search volume for face masks in different periods.**

|  | Descriptive statistics | | | Multiple comparison ^a^ | |
| --- | --- | --- | --- | --- | --- |
|  | N | Mean | SD | 2^b^ | 3^b^ |
| 1. Stage one | 27 | 4.33 | 6.87 | -40.85 (4.32) | -21.22* (1.58) |
| 2. Stage two | 27 | 45.19 | 21.47 | - | 19.64* (4.20) |
| 3. Stage three | 31 | 25.55 | 5.20 |  | - |
| Values in parentheses are bootstrap standard error. ^a^ Post hoc Scheffé test with the bootstrap method was used.  ^b^ The reference group for the multiple comparisons of mean  * The mean difference is significant at the .05 level. | | | | | |
