# Supplementary material for: Evolving public behavior and attitudes towards COVID-19 and face masks in Taiwan: A social media study
Source: PLoS One. 2021 May 20;16(5):e0251845. doi: 10.1371/journal.pone.0251845 (PMC8136722; doi:10.1371/journal.pone.0251845)
Supplement: Attachment — Submitted filename: Response to reviewers.docx [file pone.0251845.s010.docx]

**Response letter for revised Manuscript PONE-D-20-17953R1**

“*Evolving public behavior and attitudes towards COVID-19 and face masks in Taiwan: A social media study*”

Authors: *Chih-Yu Chin*, *Chang-Pan Liu*, Cheng-Lung Wang

We are sincerely grateful to the reviewer for constructive comments. We believe that these comments have helped us enhance the quality of the manuscript. We have done our best to revise as well as improve the paper according to the comments. Please see our responses to each comment as follows.

**Comment #1:** *"This is an interesting study, that used search trends using terms related to face mask in google data base. The number of searches in the google database, not necessary a reflection of number of individuals searching for that item. A particular person, who needs an item, may search it several times on the google database and it adds to the number of searches on that particular time period. This way the google trends may not provide accurate information."*

**Response #1:**

Thank you for appreciating our effort in measuring the public epidemic awareness by the behaviors of online searches for face masks. We are also grateful to the reviews for this helpful comment.

We have checked that Google Trend would eliminate repeated inquiries from the same person over a short period regarding duplicate searches. We have added this description to the revised version of the paper. Please refer to line 188-190 of page 11.

**Comment #2:** *"The authors may discuss about the search trends related to face mask during the defined period in other countries of the SE Asian region. This will give an idea that Taiwan is successful in containing the panic buying behavior through its policy, whereas other countries, were unable to do so."*

**Response #2:**

We are very thankful to the reviewer for this helpful and constructive suggestion. In this revised version of the paper, we have discussed the cross-national comparison of Google search volume of face masks, and the total confirmed cases of COVID-19.

As for country selection, we not only discuss the infected Southeast Asian countries as suggested by the reviewer but also discussed the infected countries, which are the same as Taiwan in East Asia. Therefore, data from seven countries, including Taiwan, Singapore, Malaysia, Thailand, Vietnam, South Korea, and Japan, were collected to make a cross-national comparison in the study. These countries shared common conditions that had resisted the COVID-19 epidemic for more than 30 days from the date that the countries identified the first confirmed case of COVID-19 to February 29, 2020. Moreover, the number of total confirmed cases of COVID-19 is greater than 15 on February 29, 2020. please refer to lines 193-199 of page 11.

Moreover, the cross-national comparison findings did provide another perspective on how Taiwan might be different from other countries. Moreover, when comparing various countries simultaneously, we can better understand the universal and unique patterns of online searches for face masks across nations. We have added several paragraphs in a proper section. Please refer to line 316-336 of pages 18 and 19, line 383-395 of pages 22 and 23, line 477-489 of pages 31 and 32, and 497-510 of pages 32 and 33.
