# Supplementary material for: Evolving public behavior and attitudes towards COVID-19 and face masks in Taiwan: A social media study
Source: PLoS One. 2021 May 20;16(5):e0251845. doi: 10.1371/journal.pone.0251845 (PMC8136722; doi:10.1371/journal.pone.0251845)
Supplement: Attachment — Submitted filename: 20210202_Response to reviewer.docx [file pone.0251845.s011.docx]

**Response letter for revised Manuscript PONE-D-20-17953R1**

“*Evolving public behavior and attitudes towards COVID-19 and face masks in Taiwan: A social media study*”

Authors: *Chih-Yu Chin*, *Chang-Pan Liu*, Cheng-Lung Wang

We are sincerely grateful to the reviewers for showing the approval of our manuscript and providing constructive comments. We believe that these comments have helped us enhance the quality of the manuscript. We also have done our best to revise as well as improve the paper according to the comments. Please see our responses to each comment as follows.

Reviewer 1’s comments

**Comment #1 (Reviewer 1):** *This is an interesting article. The authors had responded to all the queries raised. Revision made is satisfactory.*

**Response #1:**

Thank you for appreciating our effort in revising our previous work. We are also grateful to those constructive comments which definitely improve the quality of our manuscript.

Reviewer 2’s comments

**Comment #1:**

*I don’t have a clear sense of the argument the authors are advancing or the hypotheses they are testing. At various points the authors make claims about the effects of awareness on pandemic mitigation, but of course their design is not set up to test that possibility. Is it that COVID-19 cases caused awareness, and then mask usage for prevention?*

*Relatedly, what are the expectations related to fear and the outcomes? I get that fear may be associated with awareness and mask usage, but the authors construct some typology of fear without any clear purpose in mind. All of this needs to be much more strongly motivated by theory. I had no clear sense of what expectations the authors wanted to test, so the rest of the manuscript felt like a fishing expedition.*

**Response #1:**

We are very thankful to the reviewer for this helpful and constructive suggestion. In this revised version of the paper, we have re-construct the section of *Introduction* to make our contentions more understandable for readers. At the initial stage of the COVID-19 epidemic, Taiwan has performed a good epidemic control. However, previous research on Taiwan’s epidemic prevents seemed to discuss what measures the government has done. The study, building on previous research emphasizing the effectiveness of earlier public epidemic awareness on epidemic prevention, **provides another story about how public epidemic awareness rose and how it was associated with epidemic development, media, and government in modern society during the development of the COVID-19 epidemic**. Therefore, in this reversion, we have put more emphasis on discussing the role of media and government in the rise of public epidemic awareness. Please refer to line 73-161 from page 4 to page 9.

Moreover, regarding the reviewer’s question of what are the expectations related to fear and the outcomes, we have revised the relevant paragraphs in the section of *Introduction* and *Methods* to make our expectations related to fear and outcomes more clear.

In the section of *Introduction*, first of all, we emphasized that not only the quantity of COVID-19 related message but also the contents of the message would have an impact on the public opinion formation of COVID-19. Then, we provided theoretical and empirical evidence to emphasize the role of fear in rising public epidemic awareness and health behavior change. Finally, we acknowledged that, in this case, using fear appeals might be “The same knife cuts bread and fingers.” Thus, we then argued that understanding messages associating with various characteristics of fear spread to people and its effect on public epidemic awareness during the epidemic would be helpful for the government to enact an appropriate strategy to arouse and citizen’s fear or mitigate the panic. Thus, the study aims to explore the dynamic relationships between epidemic development and the disease-related information spread on the Internet and to explain how it contributes to the epidemic control during the early period of the COVID-19 epidemic in Taiwan. Please refer to line 107-138 from page 6 to page 8.

In the section of *Methods*, we have added the theoretical description of how each fear-arousing element makes people afraid. Please refer to line 299-309 of page 17.

**Comment #2:**

*The authors make an assumption that mask usage (or searching) can act as an indicator that the public is taking steps to protect themselves. I understand that is a reasonable assumption in Taiwan. It might be worth discussing the variation that exists on this dimension cross-nationally. We wouldn’t be able to use mask searching or usage as such an indicator when public health authorities actively dismissed their usage in many other countries until several months into the pandemic (see Merkley & Loewen, 2020).*

**Response #2:**

We are very thankful to the reviewer for this helpful and constructive suggestion. We have added some descriptions about the potential national or cultural differences in the attitudes toward the use of face masks during the COVID-19 epidemic. Also, we remind readers that it should be careful when interpreting Google search volume for fact maks as public epidemic awareness n the context of cross-country comparison. Please refer to line 441-452 of page 26.

Moreover, in the section of conclusion, we have added several sentences to acknowledged that using searching behavior of face masks as an indicator of public epidemic awareness cannot be used in all countries. However, the purpose of the study did not advocate using a universal indicator for measuring or monitoring public epidemic awareness. Instead, we argued that finding out an appropriate digital footprint of public epidemic awareness in a given scenario for a region or a country is necessary since it provides real-time information on public epidemic awareness. Please refer to line 696-702 of page 43.

**Comment #3:**

*Which brings me to a much more substantive point: government communications are notably absent from this analysis. As noted, I’m not entirely clear on the argument advanced here. But it seems like there is an assumption that cases generated media and public awareness, and subsequently searching related to masks.* *Of course, cases increased in other Asian countries, but not the same level of searching for masks, which could be in large part due to the seriousness and promptness with which their governments acted on the pandemic and the mitigation strategies they communicated to their mass publics. I think there would be a lot of value added to the analysis if there was some effort to identify a link between government communication and media/the public, along the lines of what we see in agenda setting literature (see Soroka 2002 and many others along those lines)*

**Response #3:**

We are very thankful to the reviewer for this helpful and constructive suggestion. First of all, the study aims to obtain valuable experience based on Taiwan’s response to COVID-19, involving citizens, news media, and the government. Indeed, there is an assumption that confirmed cases of COVID-19 in Taiwan generated media and public awareness, and subsequently searching related to masks. Our results also suggested that news media and the public immediately attached great importance to the imported cases of COVID-19 in Taiwan.

In the revised manuscript, we have added some discussion about the role of government communication. We emphasize that despite the matter of media on the spread of COVID-19 information, the role of government communication cannot be neglected when it comes to the rise of public epidemic awareness. We discussed how government communication in a transparent, accurate, and timely manner on epidemic control in Taiwan via holding daily press briefing. Please refer to line 574-593 from page 36 to page 37.

Moreover, in the original manuscript of the *results* section, we put much effort into a discussion about how government measures and public epidemic awareness mutually affect each other, which provides another perspective on the government-public relationships. Please refer to line 460-514 from page 27 to page 29.

**Comment #4:**

*My biggest concerns relate to the data and methods. First, much more information needs to be given about the precise nature of the data being used in this manuscript, especially related to social media. We need more information on what data KEYPO collects and from what platforms. “Social media” is a very broad term. What exactly is the structure of the data that is provided? How is it accessed? What keywords are used? What are its limitations? None of this is transparently discussed in the manuscript.*

**Response #4:**

We are very grateful to the reviewer for this helpful and constructive suggestion. Also, we are sorry that the information about the data and method in the previous version is not enough for readers to understand the research method of the study. In the revised version, we have uploaded supporting information called “Appendix S1. Information about the scope of the data and KEYPO big data analytics engine.”

In Appendix S1, we introduced the research subjects, data source, the time range of data, keywords for analysis, and techniques for retrieving data in the study. We hope this information is helpful for the reviewer to understand the methods used in the study. Please refer to Appendix S1.

**Comment #5:**

- A minor, but related point. Wikipedia is not an acceptable source for students, and is not an acceptable source for published research. These data need to be verified from the original source.

**Response #5:**

Thank you for the suggestion. Regarding the global number of confirmed cases between December 31 and January 19, we have replaced the source of Wikipedia with WHO released data (<https://covid19.who.int/).> Please refer to line 230-231 of page 13.

The results associating with the global number of confirmed cases have also been modified, including Figures 1 and 2.

**Comment #6:**

Second, I do not think the authors are taking the time series nature of their data seriously enough. There is temporal dependence between these data that do not lend themselves well to simple correlations. For example, confirmed cases will be mechanically related to prior values (in fact, these variables are unit root by definition), while others, like social media and news reports may be (trend) stationary. Autocorrelation

**Response #6:**

We are very grateful to the reviewer for this helpful and constructive suggestion. In the revised version of the manuscript, we have changed the way we analyze the relationships among time series variables, including the number of confirmed cases of COVID-19, volumes of mentions of COVID-19 and face masks, and Google search volume for COVID-19 and face masks. We adopted time series analysis (TSA) conducted with SPSS version 22 to deal with the nature of time-series data. Specifically, three functions, namely, the autocorrelation function (ACF), the partial autocorrelation function (PACF), and the cross-correlation function (CCF) were implemented. The first two functions, ACF and PACF, were used to identify the temporal dynamics of an individual time series variable and help researchers to examine whether the errors of a certain series variable would correlate itself over time. If a certain variable has autocorrelative relationships, the series data was whitened by removing the autocorrelation before conducting the CCF. The CCF was used to examine the dynamic relationship between two time series variables by calculating the correlation coefficients between two time series variables contemporaneously and at various lagged values. It gives us an insight into how two time series variables coincidently related to each other, and whether movement in one variable tends to precede or follow movement in the other. Please refer to line 264-278 of page 15. The corresponding results of time series analysis were also reported. Please refer to line 336-354 from page 19 to page 21 and line 384-394 of page 23.
