# Supplementary material for: Evolving public behavior and attitudes towards COVID-19 and face masks in Taiwan: A social media study
Source: PLoS One. 2021 May 20;16(5):e0251845. doi: 10.1371/journal.pone.0251845 (PMC8136722; doi:10.1371/journal.pone.0251845)
Supplement: Attachment — Submitted filename: 20210402_Response to reviewer.docx [file pone.0251845.s012.docx]

**Response letter for revised Manuscript PONE-D-20-17953R2**

“*Evolving public behavior and attitudes towards COVID-19 and face masks in Taiwan: A social media study*”

Authors: *Chih-Yu Chin*, *Chang-Pan Liu*, Cheng-Lung Wang

We are sincerely grateful to the reviewers for showing the approval of our manuscript and providing constructive comments. We believe that these comments have helped us enhance the quality of the manuscript. We also have done our best to revise as well as improve the paper according to the comments. Please see our responses to each comment as follows.

Academic editor’s comments

**General comments (Academic editor):** *Please review your reference list to ensure that it is complete and correct. If you have cited papers that have been retracted, please include the rationale for doing so in the manuscript text, or remove these references and replace them with relevant current references.*

**Response #1:**

We are very thankful to the editor for reminding us of checking the correctness of references. We have found that some references (news articles) need permission to browse because they are more than six months away from publication time. We have replaced them. Please see the revised references list below.

New reference lists.

[43] Everington K. No need for healthy people to constantly wear masks: Taiwan CECC. Taiwan News. 2020 Jan 31 [Cited 2020 Mar 10]. Available from: <https://www.taiwannews.com.tw/en/news/3868005>.

[47] Everington K. Taiwan platform includes over 100 apps showing mask availability in stores. Taiwan News. 2020 Feb 27 [Cited 2020 Mar 8]. Available from: https://www.taiwannews.com.tw/en/news/3882111.

[48] Everington K. Wuhan Virus: Taiwan's ban on face mask exports to last until May. Taiwan News. 2020 February 13 [Cited 2020 Mar 8]. Available from: https://www.taiwannews.com.tw/en/news/3875745.

Reviewer 3’s comments

**General comment #1 (Reviewer 3):**

(1) *I would like to suggest editing the introduction section. What the authors intend to achieve with this paper could be better aligned; clearly and succinctly reflected at the relevant place in the manuscript – at the end of the introduction.*

*3) The aims of the study could be carefully pulled together and distinct from the Introduction in lines 102-105, 132 - 137, 166 -172 and 182 -184. And the statement in 181-184 on content analysis should be reflected as an objective of the study if that was the intention.*

*Please refer to aims of the study mentioned in different sections. These are listed below. I think they can be pulled together/aligned.*

*(i) Lines 102 – 105. “Given that, one of the purposes of the study is to examine the dynamic relationships between epidemic development and the disease related information spread on the Internet, and to explain how it contributes to the epidemic control during the early period of the COVID-19 epidemic in Taiwan.”*

*(ii) Lines 132 -137 -Thus, this study aims to fill the gap in the literature on the diffusion of various fear-related messages on the Internet, including mistrust, severity, loss of control, uncertainty, and susceptibility, drawing from previous literature on fear and risk perception [14, 21-23], and their effects on public epidemic awareness along with the development of the COVID-19 epidemic.*

*(iii) Lines …166 -172………….. This naturalistic study aimed to capture public epidemic awareness of COVID-19 through collecting social media- and Internet-based data, and attempted to elaborate on how the public epidemic awareness rose, and how it played a role in contributing to the successful epidemic prevention in Taiwan during the spread of COVID-19.*

*(iv) Lines 181 – 184 … Moreover, this study attempted to analyze the transmission of different types of fear information of COVID-19 on the Internet from December 31, 2019 to March 29, 2020, and their effects on the public epidemic awareness by conducting a content analysis.*

**Response #1:**

We are very thankful to the reviewer for this helpful and constructive suggestion. Regarding the general comments (1) and (3), in this revised version of the paper, we have combined the research purposes, scattered initially in different paragraphs, at the end of the introduction and make our contentions more understandable for readers.

At the end of the Introduction section, we split the research purposes into. The first is to document real-time public epidemic awareness of COVID-19 through collecting disease-related information-seeking behaviors on Google in Taiwan and examines its dynamic relations to epidemic development, disease-related information spread on the Internet and government face-mask related policies during the initial phase of the spread of COVID-19 in Taiwan. The other is to investigate the diffusion of various fear-related messages on the Internet and their effects on public epidemic awareness along with the development of the COVID-19 epidemic. Please refer to line 173-194.

**General comment #2 (Reviewer 3):**

*2) The authors need to clarify whether this article is building on past hypotheses or examining the role of the media and government in increasing public epidemic awareness or both. The above is not clear from the introduction.*

**Response #2:**

We are very grateful to the reviewer for the reminders of unclear statements. Since the study, building on previous research emphasizing the effectiveness of earlier public epidemic awareness on epidemic prevention provides another story about **how public epidemic awareness rose and how it was associated with epidemic development, media, and government in Taiwan during the development of the COVID-19 epidemic**. Therefore, in this reversion, we add and revise some paragraphs to focus on the government’s and media’s role in arousing the public epidemic awareness.

The revised paragraphs are listed below.

Lines 67-72: “The study aims to fill in the story of Taiwan’s experience of defending against COVID-19 at an early stage from the perspectives of public epidemic awareness and the prominent role of media in the transmission of COVID-19-related information. Also, the study documents the government’s face-masks countermeasures against COVID-19 and how it associates with public epidemic awareness.”

Lines 85-93: “During a public health crisis, such as emerging infectious disease, effective health communication is vital for the rise of public epidemic awareness, where government and media play crucial roles [11, 12, 13, 14]. As for a government, it is essential to adopt an effective communication strategy with useful content, trustworthy sources, and efficient channels to enable the public to be aware of or learn about disease-related information in a timely manner. Previous research has indicated the higher public’s exposure to government information about COVID-19, the public would have higher probabilities to take necessary preventive measures to protect themselves from being infected by COVID-19 [11].”

References 11 and 12 are the newly added articles regarding the role of government communication:

[11] Chang C. Cross-country comparison of effects of early government communication on personal empowerment during the COVID-19 pandemic in Taiwan and the United States. Health Commun. <https://doi.org/10.1080/10410236.2020.1852698>

[12] Freimuth V, Linnan HW. Potter P. Communicating the threat of emerging infections to the public. Emerging Infect. Dis.2000; 6(4): 337–347.

**General comment #3 (Reviewer 3):**

*There are doubtful assumptions in this manuscript.*

***(i)*** *Awareness will most likely lead to adoption of appropriate preventive measures*

***(ii)*** *The more the media coverage and social media comments the more likely a reduction of prevalence will be achieved due to increased adoption of preventive measures by the public – without taking into consideration the contents of the social media/mass media*

***(iii)*** *Fear predisposes positive action.*

***(iv)*** *Increase in case incidence (or rather the announcement/reporting of it) during a pandemic/epidemic will lead to greater media attention and social media activity on that epidemic. It may, on a very short period. Incidentally, the authors indicated that government actions such as the banning of export of facemasks rather than the number of new cases led to rise/decrease in social media activities/mentions. These may have as well influenced adoption of preventive behaviours by the public.*

*Some of these assumptions have been acknowledged by the authors but the evidence put forward in this paper do not support most of the assumptions. It is important the authors rethink these and provide additional evidence.*

**Response #3:**

We are very thankful to the reviewer for this helpful and constructive suggestion. As the reviewer mentioned, many of our assumptions are based on previous literature rather than our provided evidence. Therefore, in this revised version, we have changed the statements, especially the sub-title, and focused on describing how Taiwan's public epidemic awareness emerged and the media and the government's role in it, trying to remove these doubtful assumptions.

Regarding the first doubtful assumption that *awareness will most likely lead to adoption of appropriate preventive measures*, we have substituted the too strong statement entitled “Rapidly increasing public epidemic awareness effectively prevent its spread” with “The role of news media and government in rapidly increasing public epidemic awareness in the early stage of the spread of COVID-19.” Please refer to lines 173-194. We also reviewed the entire content under the sub-section. We emphasize the rise of public epidemic awareness and its relations to the news media and the government in the revised version, It seems that the content is more in line with the revised titile.

As for the second doubt assumption, we are sorry that we are wondering whether we made this hypothesis. We mentioned in the introduction that when emerging infectious diseases are heavily and quickly discussed in media, the public can better prepare and prevent them earlier (in lines 99-105). However, in our results or the discussion section, we did not mention that a reduction of prevalence in Taiwan is because the people have been affected by the mass media or social media discussing COVID-19 and then have taken prevention measures. What we emphasize is the part of the rise of public epidemic awareness, so we add a sentence into the paragraph. Please refer to line 589.Moreover, , we added a paragraph about the limitation and future suggestions at the end of the discussion as below:

“Finally, our research used the web methodology to collect timely information about public epidemic awareness and the quantity of disease-information spread on the Internet and examine the dynamic relationships. However, we did not consider the content of information transmitted on the Internet. Future studies might adopt various analytics such as text mining and sentiment analysis to take what the news spread into account. Please refer to line 712-717.”

Regarding the third assumption that *fear predisposes positive action*, we have replaced the statement entitled “The role of fear in stimulating epidemic awareness” with “Types of fear-embedded popular events and public epidemic awareness in the course of COVID-19 spread in Taiwan.” Please refer to lines 672-673. We removed the sentences that comparing the loss of control is more substantial than perceived susceptibility and perceived severity to cause people to wear a face mask. Moreover, we have pointed out more euphemistically: the loss of control might explain why panic behavior and massive searching behaviors for face masks occur in Taiwan (Please refer to line 696-699).

As for the fourth assumption, we have removed the statement that “the volume of the mentions and search query of face masks became less affected by new cases of infection.” Please refer to lines 500-503.

**Specific comment #4 (Reviewer 3):**

*Lines 22 – 23: “This study aims to capture public epidemic awareness of COVID-19 through collecting social media” – ‘document’ maybe a better word to use rather than ‘capture”.*

**Response #4:**

Thank you for choosing a better word in this context. We have replaced the word “capture” with “document.” Please refer to line 23 and 172.

**Specific comment #5 (Reviewer 3):**

*Lines 24 – 26: … “and elaborating on how public epidemic awareness rose and played a role in epidemic prevention in Taiwan during the initial phase of the spread of COVID-19”. This needs to be rephrased. How "awareness" played a role in epidemic prevention (i.e. risk mitigation) is not sufficiently documented in the paper.*

**Response #5:**

We are very thankful to the reviewer for this helpful and constructive suggestion. We have modified the purpose of this study to make it more representative of our research. The revised sentences are following:

“This study aims to document public epidemic awareness of COVID-19 in Taiwan through collecting social media- and Internet-based data, and provide valuable experience of Taiwan’s response to COVID-19, involving citizens, news media, and the government, to aid the public in overcoming COVID-19, or infectious diseases that may emerge in the future.” Please refer to lines 24-27.

**Specific comment #6 (Reviewer 3):**

*Lines 40 – 41: “Moreover, online social media promptly reflect public epidemic awareness, which can be used as a reference for epidemic prevention”. This should be rephrased to indicate the social media activities could be a reflection of public epidemic awareness which can be harnessed for epidemic control.*

**Response #6:**

We are very thankful to the reviewer for this helpful and constructive suggestion. We have rephrased the sentence as below:

“Moreover, in the era of digitalization, online and social media activities could reflect public epidemic awareness which can e harnessed for epidemic control.” Please refer to lines 41-42.

**Specific comment #7 (Reviewer 3):**

*Lines 58 – 60: “Combining various viewpoints to look at the effectiveness of COVID-19 prevention in Taiwan might be instructive for other countries in dealing with the next crisis resulting from emerging infectious diseases.” This conclusion is not based by the data. Perhaps, the essence of this paper should be on assessing* *information-seeking by the public during the COVID-19 pandemic.*

**Response #7:**

We are very thankful to the reviewer for this helpful and constructive suggestion. We have rewritten the sentences to a better representative of our research as follows:

“The study aims to fill in the story of Taiwan’s experience of defending against COVID-19 at an early stage from the perspectives of public epidemic awareness measured by disease-related information-seeking by the public and the prominent role of media in the transmission of COVID-19-related information. Also, the study documents the government’s face-masks countermeasures against COVID-19 and how it associates with public epidemic awareness. This might be instructive for other countries in dealing with the next crisis resulting from emerging infectious diseases.” Please refer to lines 67-74.

**Specific comment #8 (Reviewer 3):**

*Lines 67 – 69: ‘However, COVID-19 is more contagious than severe acute respiratory syndrome (SARS) and is infectious even through asymptomatic carriers’ – while this is not in doubt one wonders why this comparison is coming up without previous reference to SARS in this paragraph. A simple sentence on the fact that COVID-19 is very contagious will be helpful.*

**Response #8:**

Thank you for suggesting a better way to express the core idea. We have replaced it with a simple sentence as the reviewer suggested as follows:

“However, COVID-19 is very contagious and is infectious even through asymptomatic carriers [7]” Please refer to lines 64-65.

**Specific comment #9 (Reviewer 3):**

*Lines 72 – 73: ‘Public epidemic awareness has been recognized as a crucial determinant of public epidemic prevention’ – this needs to be supported by literature*

**Response #9:**

Thank you for reminding us to add some references. Please refer to line 76.

**Specific comment #10 (Reviewer 3):**

*Lines 76 – 79: ‘Recent research on public health awareness during the COVID-19 epidemic also showed that one’s awareness of how to prevent COVID-19 significantly contributes to one’s behavioral chance of fighting against COVID-19 [10]’. Only one article is cited to support this statement. I recommend the statement be modified to reflect this e.g. citing the publication specifically.*

**Response #10:**

Thank you for the reminder. We have added “e.g.,” before the reference [10].

**Specific comment #11 (Reviewer 3):**

*Lines 166 – 188: These lines can better be summarized and synchronized with information in other parts as to what the study intends to achieve.*

**Response #11:**

We are very thankful to the reviewer for this helpful and constructive suggestion. We have combined the purpose of this study into the end of the introduction to make better aligned. Please refer to lines 173-194.

**Specific comment #12 (Reviewer 3):**

*Lines 218 -225. Justify the selection of two out of seven indicators (volume of mentions of COVID 19 and the volume of mentions of face masks) as indicators of public epidemic awareness. Lines 301 – 302: Some repetition - edit*

**Response #12:**

We are very thankful to the reviewer for this helpful and constructive suggestion. In this reversion, we added a paragraph in the Method section to justify the Google search volume for COVID-19 and face masks as indicators of public epidemic awareness.

First of all, disease information-seeking behaviors have been used as an indicator of public epidemic awareness. Moreover, we argued that increasing intended behaviors of searching for face masks indicates that the public is aware of the threat and reflects other positive hygiene practices. These are the reasons why we choose the two variables to reflect public epidemic awareness. The paragraph shows below:

“The disease information searching behaviors have been used as an indicator of public epidemic awareness recently [29]. It reflects the behavioral intentions of individuals. In addition to searching for disease, given that related treatment drugs and vaccines for COVID-19 were under clinical trials in the early stage of COVID-19 [33], face masks have become necessary personal protective equipment for managing infectious diseases [34,35]. Increasing intended behaviors of searching for face masks indicates that the public is aware of the threat and reflects other positive hygiene practices [36,37]. Thus, we examine the public epidemic awareness by measuring the public online information-seeking behavior of COVID-19 and face masks.” Please refer to lines 231-240.

**Specific comment #13 (Reviewer 3):**

*Lines 301 – 302: Some repetition - edit*

**Response #13:**

Think you for the kindly reminder. The repetition has been edited. Please refer to line 316.

**Specific comment #14 (Reviewer 3):**

*Lines 488 -491. However, the concluding statement in lines 488-491 “Such positive actions reduced public anxiety about not being able to buy face masks; accordingly, the volume of the mentions and search query of face masks dropped significantly and became less affected by new cases of infection” needs a reference or a supporting data of this study to justify the claim that a “search query of face masks dropped significantly and became less affected by new cases of infection”*

**Response #14:**

We are very thankful to the reviewer for this helpful and constructive suggestion. Since our evidence indeed cannot prove that “the volume of the mentions and search query of face masks became less affected by new cases of infection,” we have removed the statement. Please refer to lines 500-503.

**Specific comment #15 (Reviewer 3):**

*Lines 497-500. I am not convinced the data of this study justifies the authors conclusion that masks prevent epidemic of fear —see authors concluding statement “…masks, thus preventing an epidemic of fear.”*

**Response #15:**

We are very thankful to the reviewer for this helpful and constructive suggestion. After we reviewed the sentence, we removed the sentence “this preventing an epidemic of fear.” We agree with the reviewer that it is explained to an excessive degree. Please refer to line 512.

**Specific comment #16 (Reviewer 3):**

*Lines 509 – 510: ‘It should be noted that keeping the public epidemic awareness at a high level might be crucial for combating COVID-19.’ This, in my opinion, is the best conclusion of this study.*

**Response #16:**

Thank you for appreciating our argument.

**Specific comment #17 (Reviewer 3):**

*Lines 516 -517. This is unclear, please explain “According to the ANOVA results (Table 2), the types of fear had a significant effect on the Google search volume of face masks [F(3,358) = 5.67, p < .05].”*

**Response #17:**

Thank you for the reminder of the unclear statement. We have explained the results that the Google search volume for face masks varied by the dissemination of various types of fear-embedded messages. Please refer to lines 530-531.

**Specific comment #18(Reviewer 3):**

*Lines 554 – 555: I think this is an overgeneralization. There are other factors that contribute.”*

**Response #18:**

We are very thankful to the reviewer for this helpful and constructive suggestion. After careful consideration, we agree with the reviewer's opinion that it is an overgeneralization. Thus, we have substituted the too strong statement entitled “Rapidly increasing public epidemic awareness effectively prevent its spread” with “The role of news media and government in rapidly increasing public epidemic awareness in the early stage of the spread of COVID-19.” Please refer to lines 173-194.

**Specific comment #18(Reviewer 3):**

*Lines 693 – 695 essentially the same as lines 703 – 705. I suggest to edit*

**Response #18:**

Think you for kindly reminder. The repetition has been edited. Please refer to lines 727-729.

Reviewer 4’s comments

**General comment #1:**

*The authors have done a fine job in revising and enhancing their manuscript and completing their shortcomings. I enjoyed reviewing the manuscript. My only comment is that although the authors' data doesn't cover practice among the general population, I was wondering if it was possible if they could add a paragraph in the discussion section based on literature regarding the practice towards face masks during their study period and whether it was inline with their evidence?*

**Response #1:**

Thank you for appreciating our effort in revising our previous work. We are also grateful for those constructive comments which definitely improve the quality of our manuscript.

Regarding the suggestion of adding a paragraph about the practice towards face masks during their study period and whether it was inline with their evidence, we have added some references to show the effectiveness of using face makes and how Taiwan strictly enforce the policy of wearing masks in Taiwan. The added paragraph shows below:

“While our data could not directly reflect one’s actual preventive behavior of wearing a face mask and prove its effectiveness of epidemic prevention, previous literature has documented that Taiwan adopted widespread measures for the public to wear masks would be the reason for the reduction of COVID-19 transmission and even having good control of the COVID-19 without a mandatory suspension of work and school [61]. For example, TCDC requires people to wear masks in public places, including public transport and hospital, and that will be fined if they fail to comply. The other research also showed that Taiwan, a mask-wearing country, has lower growth rates of COVID-19 cases compared to the non-mask-waring counties in the early stage of the spread of COVID-19 via combining mathematical modeling and existing scientific evidence [44].” Please refer to lines 648-658.

References 61 are the newly added article regarding the effectiveness of using face-masks to prevent COVID-19 spread.

[61] Wei J. Guo S. Long E. Zhang L. Shu B. Guo L. Why does the spread of COVID-19 vary greatly in different countries? Revealing the efficacy of face masks in epidemic prevention. Epidemiol. Infect. 2021. https://doi.org/10.1017/S0950268821000108
